# Clinicopathological features of the nasopalatine duct cyst: A systematic review

**DOI:** 10.1007/s10006-026-01515-x

**Published:** 2026-02-21

**Authors:** Alini Cardoso Soares, Gabriel Lima Braz, Amanda dos Santos Figueiredo, Camila Barcellos Calderipe, Francisco Samuel Rodrigues Carvalho, Fábio Wildson Gurgel Costa, Ana Carolina Uchoa Vasconcelos

**Affiliations:** 1 Department of Oral Diagnosis, Piracicaba Dental School, University of Campinas- UNICAMP, Piracicaba, São Paulo Brazil; 2 Postgraduate Program in Dentistry, Dental School, Federal University of Pelotas- UFPel, Pelotas, Rio Grande do Sul Brazil; 3 Department of Oral Pathology, School of Dentistry, Federal University of Rio Grande do Sul- UFRGS, Porto Alegre, Brazil; 4 Graduate Program in Health Sciences, Federal University of Ceará, Sobral Campus, Sobral, Brazil; 5 Department of Clinical Dentistry, Faculty of Pharmacy, Dentistry and Nursing. Postgraduate Program in Dentistry, Federal University of Ceará - UFC, Fortaleza, Ceará Brazil; 6 Department of Operative Dentistry, Faculty of Pharmacy, Dentistry and Nursing, Postgraduate Program in Dentistry, Federal University of Ceará - UFC, Fortaleza, Ceará Brazil

**Keywords:** Jaw cysts, Nonodontogenic cysts, Non-odontogenic lesions, Nasopalatine duct cyst

## Abstract

**Purpose:**

This systematic review aimed to synthesize the available clinicopathological data on nasopalatine duct cyst (NPDC).

**Methods:**

Electronic searches were performed in seven databases and the grey literature, according to the PRISMA 2020 statement. Risk of bias was assessed using the Joanna Briggs Institute critical appraisal tool. Data were analysed using descriptive statistics.

**Results:**

A total of 168 studies comprising 2,734 NPDC cases were included. Most lesions were asymptomatic (*n* = 358/54.16%) and occurred in males (*n* = 1641/60.75%), with individuals in the fourth and fifth decade of life being the most frequently affected. Periapical radiographs (*n* = 181/40.31%) and computed tomography scan (*n* = 159/35.41%) were the most commonly used imaging modalities. Metaplasia (*n* = 278/58.28%) was observed in most cases with a predominance of combinations involving stratified squamous epithelium and pseudostratified ciliated columnar epithelium (*n* = 78/47.27%). The periapical cyst was the second most frequent clinical diagnostic hypothesis. Enucleation was the preferred treatment modality (*n* = 544/95.94%), and recurrence was absent in most cases (*n* = 375/93.05%).

**Conclusion:**

NPDC is a benign lesion with a heterogeneous clinical presentation and a low recurrence rate. Its anatomical location frequently results in diagnostic overlap with odontogenic lesions, particularly periapical chronic lesions. Clinicians should be aware of its clinicopathological features and integrate clinical, radiographic, and histopathological findings to ensure accurate diagnosis and appropriate management.

**Supplementary information:**

The online version contains supplementary material available at 10.1007/s10006-026-01515-x.

## Introduction

The nasopalatine duct cyst (NPDC) is the most common non-odontogenic lesion (NOL) of the maxillofacial region, with reported prevalence rates ranging from 2.2% to 32.8% in observational studies [1]. The aetiology of NPDC remains a subject of debate, with two main hypotheses proposed: (1) activation of epithelial remnants of the embryonic nasopalatine duct in response to irritative stimuli, such as local trauma or infections [2, 3]; and (2) spontaneous epithelial proliferation in the absence of any apparent triggering factor [4, 5].

NPDC typically presents as an asymptomatic, well-defined radiolucent or hypodense lesion in the anterior maxilla [6]. Due to overlapping clinical and radiographic features with other odontogenic and non-odontogenic lesions, including periapical cysts, and enlarged incisive foramen, accurate diagnosis is crucial for appropriate management [3, 7]. Histologically, approximately 90% of NPDCs are lined by non-keratinised stratified squamous epithelium, with focal areas of cuboidal, columnar or ciliated epithelium. Additionally, salivary gland tissue and cartilaginous remnants may be identified within the cyst wall [6, 8].

A substantial proportion of the available information on NPDC derives from isolated primary studies, mainly case reports and case series, which limits the generalisability of current knowledge. This underscores the need for a more comprehensive understanding of this entity. Therefore, the aim of this study was to review the existing literature on NPDC to address the following question: what are the clinical, demographic, imaging and histopathological features of this lesion?

## Materials and methods

### Eligibility criteria

The inclusion criteria were based on the acronym PECOS (Population, Exposure, Comparison, Outcomes, Study Design), as follows:

- P) patients of any age;
- E) presence of a histopathological diagnosis of NPDC;
- C) not applicable (no comparison group required);
- O) clinical, demographic, imaging and histopathological characteristics of patients with a confirmed diagnosis of NPDC;
- S) observational studies.

Eligible studies included patients of all ages and both sexes diagnosed with NPDC, with confirmation based on histopathological evaluation. Exclusion criteria were as follows: (1) Studies in which the reported lesion was not NPDC; (2) Reviews, book chapters, letters to the editor, personal or expert opinions, conference abstracts; experimental or in vitro studies; (3) Studies for which the full-text was not available; (4) Duplicate records identified across the main databases and the grey literature.

### Information sources and search strategies

Electronic searches were conducted in October 2025 without restrictions on publication date or language in the following databases: PubMed/MEDLINE (National Library of Medicine), Scopus (Elsevier), Embase (Elsevier), Web of Science (Clarivate Analytics), Ovid MEDLINE (Wolters Kluwer), LILACS (Virtual Health Library) and Cochrane Central Register of Controlled Trials (Cochrane Library). Gray literature was also searched in Google Scholar and ProQuest. Personalized search strategies were developed for each bibliographic database **(Supplementary Table **1**)**. Additionally, a manual search of bibliographies and reference lists of selected studies was performed to identify additional publications that may have been missed in the electronic searches. All retrieved references were imported into Rayyan software ^®^ (Qatar Foundation, State of Qatar), where duplicates were removed after identification.

### Selection process

The titles and abstracts of all articles retrieved from the searches were screened independently by two authors (A.S.F. and A.C.S.). Reviewer calibration was performed by assessing the agreement between the two reviewers when evaluating the titles and abstracts of the first 50 references identified in the searches. A kappa coefficient of 0.90 indicated excellent inter-reviewer agreement. After calibration, the two authors proceeded with the screening of all references. If the title and abstract met the inclusion criteria, the article was selected for full-text assessment. Full texts were also obtained for articles whose titles and abstracts did not provide sufficient information for a definitive eligibility decision. After full-text evaluations, studies that satisfied the eligibility criteria were included. Divergent opinions regarding inclusion or exclusion between A.S.F. and A.C.S. were resolved through discussions with a third reviewer (G.L.B.).

### Data extraction

Data were extracted from each included article, when available, as follows: patient age (in years), sex (female or male), presence or absence of symptoms, and clinical presentation (nodule, papule, or mass). Information on the initial clinical diagnosis (e.g., nasopalatine duct cyst, periapical cyst, dentigerous cyst, giant cell granuloma, among others), pulp condition (vital, necrotic, or not reported), time of evolution (in months), and treatment approach (enucleation, decompression, or marsupialisation) was collected.

Imaging variables included the type of imaging used (periapical radiographs, computed tomography scan [CT], magnetic resonance imaging [MRI], ultrasound, or combinations of these exams), delimitation (well-defined or ill-defined), locularity (unilocular or multilocular), radiodensity (radiolucent/radiopaque, hyperdense/hypodense, hyperintense signal/hypointense signal, or mixed), lesion shape (oval, heart-shaped, round, pear-shaped), and lesion size (in centimeters, when available). Data on root resorption were also recorded.

Histopathological features encompassed the epithelial lining type (stratified squamous, pseudostratified ciliated columnar, simple cuboidal, simple columnar, or mixed epithelial linings within the cyst wall), and components of the cyst wall such as neurovascular bundles, salivary gland tissue, and hyaline cartilage. Recurrence (yes/no) and follow-up duration (in months) were also included.

### Study risk of bias assessment

The Joanna Briggs Institute (JBI) tool for case reports and case series was employed to evaluate the included articles [9]. Each case report was assessed across eight parameters, and each case series across seven parameters, with all items classified as “yes” (low risk of bias), “no” (high risk of bias), or “not applicable”. The specific parameters used for each study design are detailed in **Supplementary Tables **2** and **3.

Two reviewers independently performed the risk-of-bias assessments, with disagreements resolved through discussion or consultation with a third reviewer. Risk-of-bias judgments were incorporated into the interpretation of the findings; studies with higher risk of bias were interpreted with greater caution. As no meta-analysis was conducted, these assessments were used descriptively to contextualise the reliability and strength of the available evidence. Risk-of-bias data were recorded using standardized extraction sheets developed for this review.

### Data synthesis

Statistical analyses were performed using the Statistical Package for the Social Sciences (SPSS) for Windows, version 25.0 (IBM Corporation, Armonk, NY). Descriptive statistics, including means, standard deviations (SDs), frequencies, and percentages, were used to summarize the extracted data. No meta-analysis was conducted due to the heterogeneity of study designs and reporting formats.

## Results

### Study selection

The search strategy identified 976 records from seven electronic databases and an additional 205 records through other sources, including Google Scholar, ProQuest, and reference lists, totaling 1,181 references. After automatic and manual removal of 446 duplicates, 735 unique records remained for screening.

**Fig. 1:**
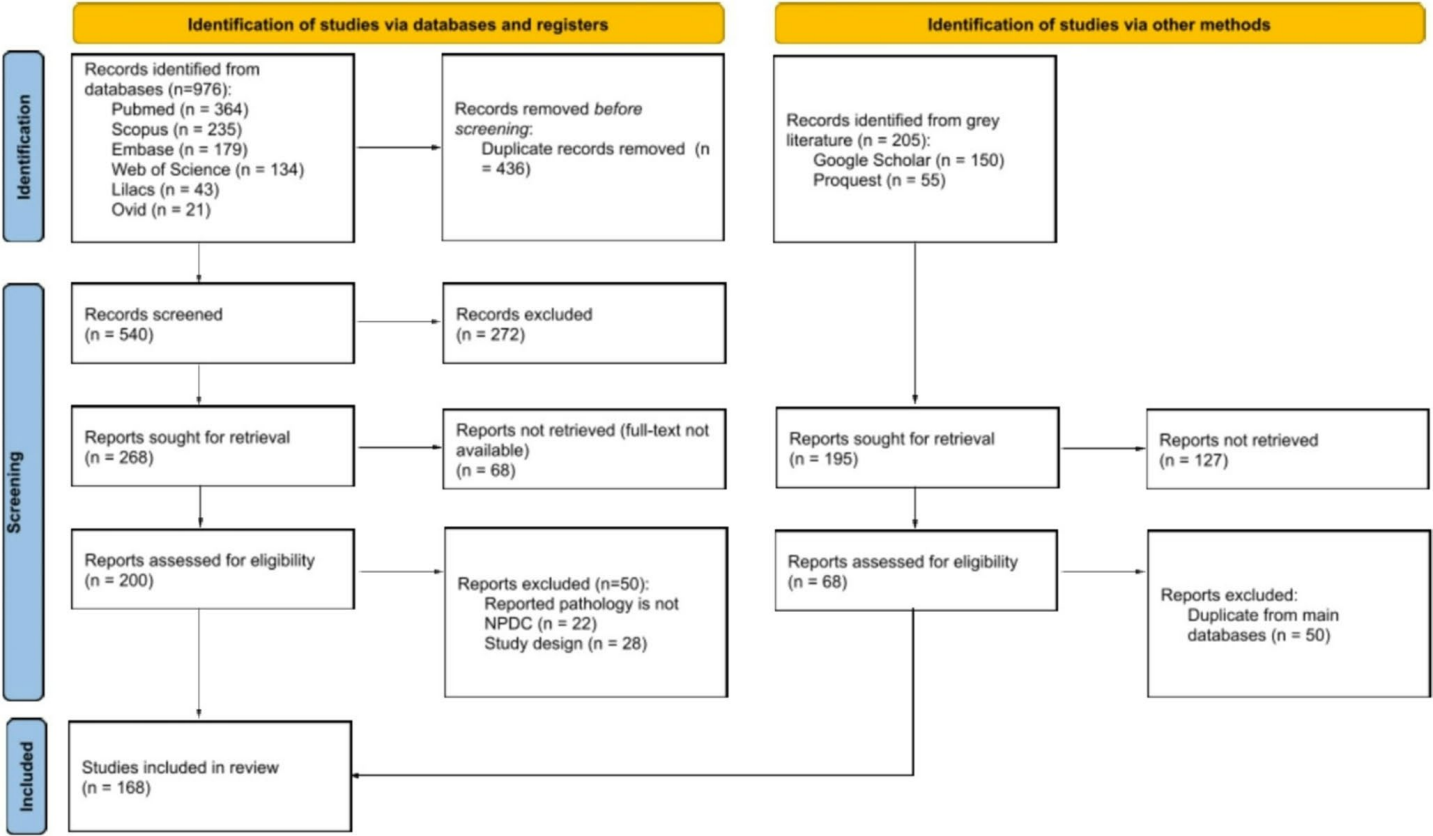
Flow diagram of literature search from PRISMA (2020)

After title and abstract screening, 268 records identified through database searches and 195 studies from other sources were selected for full-text assessment. However, 68 and 127 of these reports, respectively, could not be retrieved. Consequently, 200 reports from database searches and 68 from other sources were assessed for eligibility. Of these, 100 records were excluded due to the following reasons: pathology is not NPDC (*n* = 22), inappropriate study design (*n* = 28), or duplicate within the main databases (*n* = 50) **(Supplementary Table **4**)**. Ultimately, 168 studies, encompassing 2,734 NPDC cases, were included. The search and selection process is detailed in the PRISMA flowchart **(**Fig. 1**)**.

### Study characteristics

**Fig. 2:**
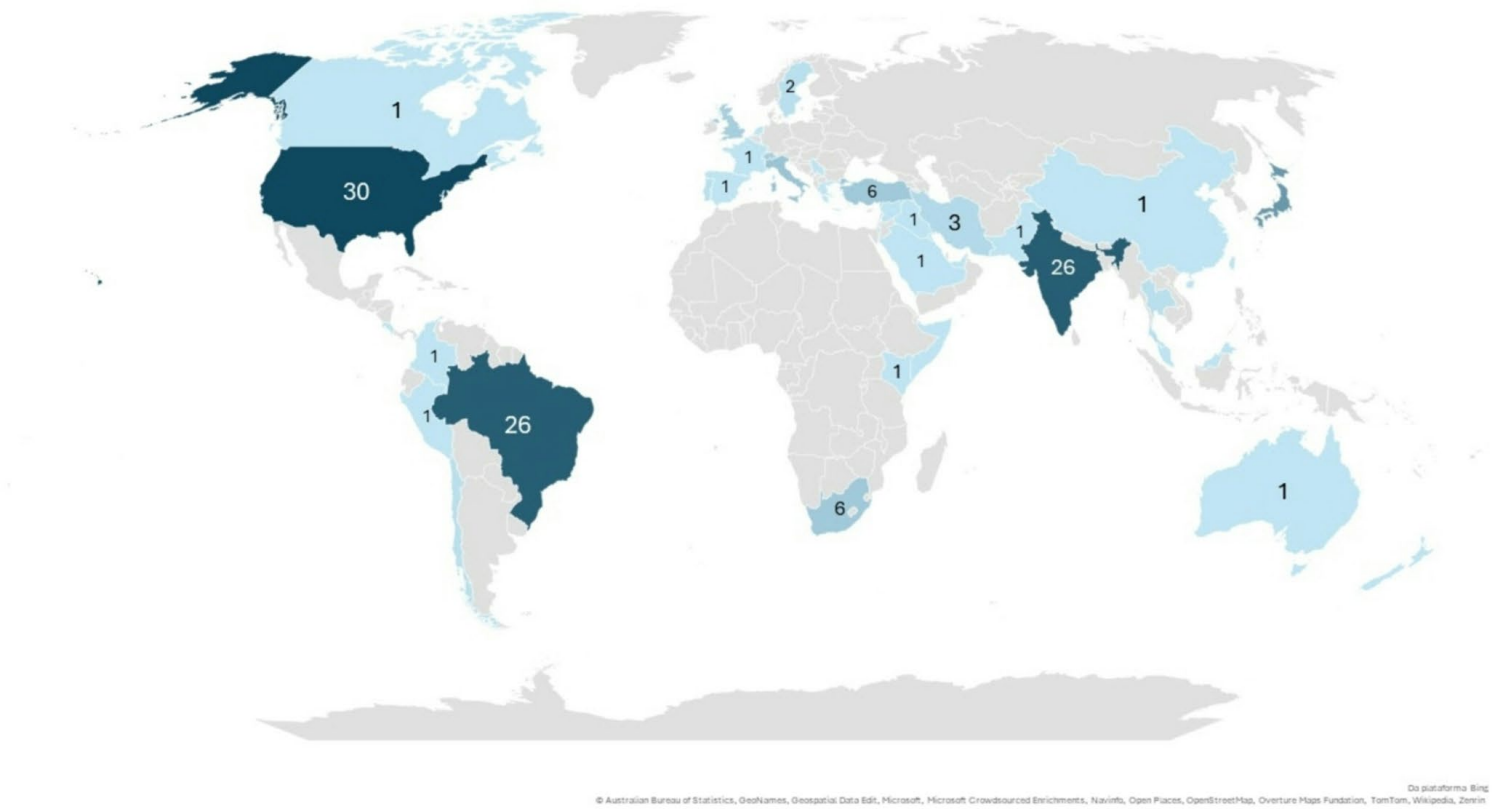
Global distribution of the articles included in the systematic review, according to country of origin

Demographic and clinical data are summarised in Table 1. The included studies originated from all continents, representing a total of 38 distinct countries. Most studies originated from Asia (*n* = 68/40.48%), followed by America (*n* = 63/37.50%), Europe (*n* = 27/16.07%), Africa (*n* = 8/4.76%), and Oceania (*n* = 2/1.19%) (Fig. 2**)**. The majority of patients were male (*n* = 1641/60.75), and the mean age at diagnosis was 36.98 ± 15.70 years (range: 7–77 years). The highest prevalence was observed among individuals in the fourth (*n* = 37/24.67%) and fifth (*n* = 31/20.67%) decades of life. Most patients were asymptomatic (*n* = 358/54.16%). The most commonly reported clinical presentation was a nodule (*n* = 461/92.75%). The most commonly reported pulpal status was that of vital teeth (*n* = 166/64.59%). Regarding clinical diagnosis, most cases were diagnosed as NPDC (*n* = 2128/98.56%), followed by periapical cyst (*n* = 14/0.64%). Mean time of evolution was 13.89 ± 21.11 months (range: 0.2–120.0 months).

**Table 1:** Demographic and clinical characteristics of the sample

Variable	Cases*		Series		Total	
Continent (*n* = 168)**	*n*	(%)	*n*	(%)	*n*	(%)
Asia	57	(44.53)	11	(27.50)	68	(40.48)
America	49	(38.29)	14	(35.00)	63	(37.50)
Europe	18	(14.06)	9	(22.50)	27	(16.07)
Africa	3	(2.34)	5	(12.50)	8	(4.76)
Oceania	1	(0.78)	1	(2.50)	2	(1.19)
Sex (*n* = 2701)						
Male	106	(70.67)	1535	(60.17)	1641	(60.75)
Female	44	(29.33)	1016	(39.83)	1060	(39.25)
Male-to-female ratio	1.4–3.4		1.6–2.5		1.6–2.5	
Age (*n* = 149)						
Mean (SD)	36.98	(± 15.70)	-		36.98	(± 15.70)
Range	7–77 years		-		7–77 years	
Symptoms (*n* = 661)						
Asymptomatic	80	(64.00)	278	(51.86)	358	(54.16)
Symptomatic	45	(36.00)	258	(48.14)	303	(45.84)
Clinical presentation (*n* = 497)						
Nodule	105	(82.68)	356	(96.22)	461	(92.75)
Absent	17	(13.38)	0	(0.00)	17	(3.42)
Papule	1	(0.79)	10	(2.70)	11	(2.22)
Mass	4	(3.15)	4	(1.08)	8	(1.61)
Pulp Diagnosis (*n* = 257)						
Vital	47	(56.62)	119	(68.39)	166	(64.59)
Necrosis	15	(18.08)	32	(18.39)	47	(18.29)
Not associated teeth	21	(25.30)	23	(13.22)	44	(17.12)
Clinical Diagnosis (*n* = 2159)***						
Nasopalatine duct cyst	97	(77.60)	2031	(99.85)	2128	(98.56)
Periapical cyst	14	(11.20)	0	(0.00)	14	(0.64)
Dentigerous cyst	2	(1.60)	3	(0.15)	5	(0.23)
Central giant cell granuloma	3	(2.40)	0	(0.00)	3	(0.14)
Odontogenic keratocyst	3	(2.40)	0	(0.00)	3	(0.14)
Ameloblastoma	2	(1.60)	0	(0.00)	2	(0.09)
Asymptomatic apical periodontitis	1	(0.80)	0	(0.00)	1	(0.05)
Nasoalveolar cyst	1	(0.80)	0	(0.00)	1	(0.05)
Residual odontogenic cyst	1	(0.80)	0	(0.00)	1	(0.05)
Median palatine cyst	1	(0.80)	0	(0.00)	1	(0.05)
Time of evolution (*n* = 78)						
Mean (SD)	13.89	(± 21.11)	-		13.89	(± 21.11)
Range	0.2–120 months		-		0.2–120 months	
Treatment (*n* = 567)						
Enucleation	133	(89.86)	411	(98.09)	544	(95.94)
Marsupialization	14	(9.46)	8	(1.91)	22	(3.88)
Decompression	1	(0.68)	0	(0.00)	1	(0.18)
Follow-up (*n* = 81)						
Mean	14.56	(± 15.96)	-		14.56	(± 15.96)
Range	0.2–120 months		-		0.2–120 months	
Recurrence (*n* = 403)						
No	78	(100.00)	297	(91.38)	375	(93.05)
Yes	0	(0.00)	28	(8.62)	28	(6.95)

*SD* standard deviation * Includes case reports and case series that reported clinical data on an individual basis

** considering the number of included articles, rather than the number of cases. ***In some cases, more than one clinical hypothesis was considered

**Fig. 3:**
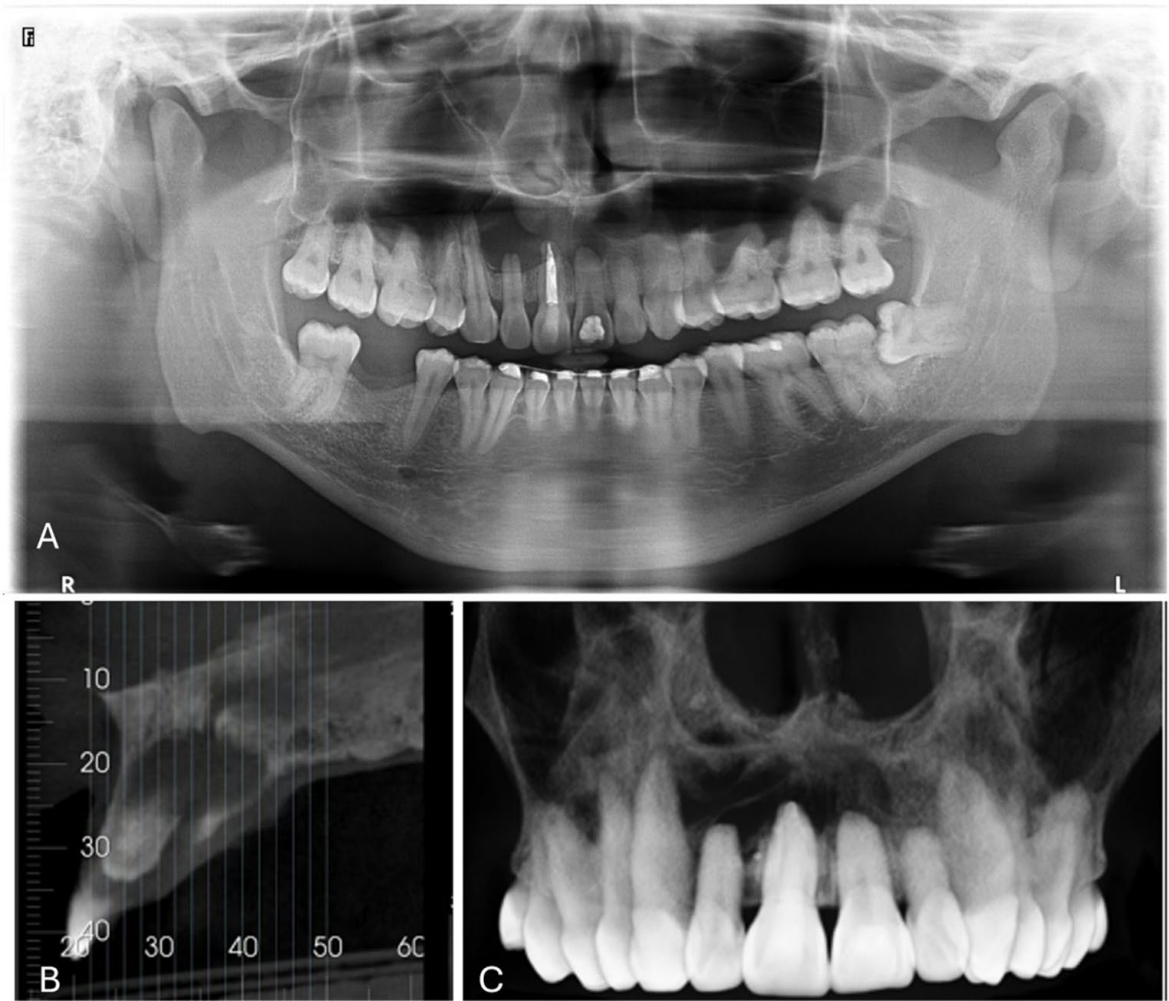
Imaging findings of the anterior maxillary lesion. (**A**) Panoramic radiograph demonstrating a well-defined radiolucent cystic lesion in the anterior maxilla, extending from the region of the right canine (tooth 13) to the left central incisor (tooth 21). (**B**) Sagittal cone-beam computed tomography (CBCT) slice showing the close anatomical relationship between the lesion and the nasopalatine canal. (**C**) Three-dimensional CBCT reconstruction highlighting the hypodense lesion and apical remodeling of the adjacent teeth

Imaging data are summarized in Table 2. Regarding imaging diagnosis, the majority of patients underwent periapical radiographs (*n* = 181/40.31%). Most lesions were described as well-defined (*n* = 512/95.52%), unilocular (*n* = 397/100.00%), oval-shaped (*n* = 222/58.27%), radiolucent or hypodense (*n* = 687/98.00%) areas. The mean size of lesions was 2.59 ± 1.57 cm (range: 0.4–7.0 cm). Root resorption was absent in 50 (75.75%) cases and present in 16 (24.35%) cases (Fig. 3). Enucleation (*n* = 544/95.94%) was the preferred treatment, followed by marsupialization (*n* = 22/3.88%) and decompression (*n* = 1/0.18%). The mean follow-up period was 14.56 ± 15.96 months (range: 0.2–120.0 months). Recurrence was absent in 375 (93.05%) cases and present in 28 (6.95%) cases.

**Table 2:** Imaging features of the sample

Variable	Cases*		Series		Total	
Imaging technique (*n* = 449)	*n*	(%)	*n*	(%)	*n*	(%)
Periapical radiographs	54	(38.84)	127	(40.97)	181	(40.31)
Computed Tomography Scan (CT Scan)	20	(14.39)	139	(44.84)	159	(35.41)
Periapical radiographs and CT Scan	58	(41.73)	44	(14.19)	102	(22.72)
Magnetic Resonance Imaging (MRI)	5	(3.60)	0	(0.00)	5	(1.11)
Ultrasonographic	2	(1.44)	0	(0.00)	2	(0.45)
Delimitation (*n* = 536)						
Well-defined	144	(99.31)	368	(94.12)	512	(95.52)
Ill-defined	1	(0.69)	23	(5.88)	24	(4.48)
Locullarity (*n* = 397)						
Unilocular	142	(100.00)	255	(100.00)	397	(100.00)
Density (*n* = 701)						
Radiolucent/Hypodense	141	(94.63)	546	(98.91)	687	(98.00)
Hypersinal	8	(5.37)	0	(0.00)	8	(1.14)
Mixed	0	(0.00)	6	(1.09)	6	(0.86)
Shape (*n* = 381)						
Oval	90	(66.18)	132	(53.88)	222	(58.27)
Pear	2	(1.48)	60	(24.49)	62	(16.27)
Heart-shaped	11	(8.08)	51	(20.81)	62	(16.27)
Round	33	(24.26)	2	(0.82)	35	(9.19)
Size of lesion (*n* = 100)						
Mean (SD)	2.59	(± 1.57)	-		2.59	(± 1.57)
Range	0.4–7 cm		-		0.4–7 cm	
Root resorption (*n* = 66)						
Absent	50	(75.75)	-	-	50	(75.75)
Present	16	(24.25)	-	-	16	(24.25)

* Includes case reports and case series that reported clinical data on an individual basis

Microscopic features are summarized in Table 3. Of the 2,734 NPDC cases, 481 (17.59%) reported the presence of different types of epithelial lining, classified as either mixed or single epithelial linings. Most of these cases showed squamous metaplasia, totaling 278 occurrences (58.28%), with a predominance of combinations involving stratified squamous with pseudostratified ciliated columnar epithelium (*n* = 78/47.27%), followed by stratified squamous with pseudostratified ciliated columnar and simple cuboidal epithelium (*n* = 35/21.21%), and stratified squamous with simple cuboidal epithelium (*n* = 21/12.73%). Among the 199 cases (10.31%) with a single epithelial lining, the most frequent type was again stratified squamous epithelium (*n* = 65/57.01%), followed by pseudostratified ciliated columnar (*n* = 40/35.08%), simple cuboidal (*n* = 7/6.15%), and simple columnar (*n* = 2/1.76%). Analysis of the cyst wall revealed the presence of neurovascular bundles in 229 cases (95.82%), salivary glands in 79 cases (79.80%), and hyaline cartilage in 29 cases (60.42%).

**Table 3:** Microscopic features of the sample

Variable	Cases*		Series		Total	
Epithelium (*n* = 481)	*n*	(%)	*n*	(%)	*n*	(%)
Mixed epithelial linings **	*62*	*(48.43)*	*216*	*(61.89)*	*278*	*(58.28)*
Stratified squamous + Pseudostratified ciliated columnar	30	(48.39)	48	(46.60)	78	(47.27)
Stratified squamous + Pseudostratified ciliated columnar + Simple cuboidal	7	(11.29)	28	(27.18)	35	(21.21)
Stratified squamous + Simple cuboidal	5	(8.06)	16	(15.53)	21	(12.73)
Pseudostratified ciliated columnar + Simple cuboidal	2	(3.23)	6	(5.84)	8	(4.85)
Simple cuboidal + Simple columnar	5	(8.06)	1	(0.97)	6	(3.64)
Stratified squamous + Pseudostratified ciliated columnar + Simple cuboidal + Simple columnar	5	(8.06)	0	(0.00)	5	(3.03)
Stratified squamous + Simple cuboidal + Simple columnar	3	(4.84)	1	(0.97)	4	(2.42)
Pseudostratified ciliated columnar + Simple columnar	2	(3.23)	1	(0.97)	3	(1.82)
Stratified squamous + Pseudostratified ciliated columnar + Simple columnar	1	(1.61)	2	(1.94)	3	(1.82)
Stratified squamous + Simple columnar	2	(3.23)	0	(0.00)	2	(1.21)
Single epithelial linings **	*66*	*(43.13)*	*133*	*(8.36)*	*199*	*(10.31)*
Stratified squamous	30	(45.46)	35	(72.91)	65	(57.01)
Pseudostratified ciliated columnar	31	(46.97)	9	(18.75)	40	(35.08)
Simple cuboidal	5	(7.57)	2	(4.17)	7	(6.15)
Simple columnar	0	(0.00)	2	(4.17)	2	(1.76)
Cyst wall (*n* = 386)						
Neurovascular bundle (*n* = 239)						
Present	55	(84.62)	174	(100.00)	229	(95.82)
Absent	10	(15.38)	0	(0.00)	10	(4.18)
Salivary glands (*n* = 99)						
Present	18	(47.37)	61	(100.00)	79	(79.80)
Absent	20	(52.63)	0	(0.00)	20	(20.20)
Hyaline cartilage (*n* = 48)						
Present	8	(29.63)	21	(100.00)	29	(60.42)
Absent	19	(70.37)	0	(0.00)	19	(39.58)

* Includes case reports and case series that reported clinical data on an individual basis. ** Number of cases that reported the presence of multiple or a single epithelial type

### Risk of bias in studies

**Fig. 4:**
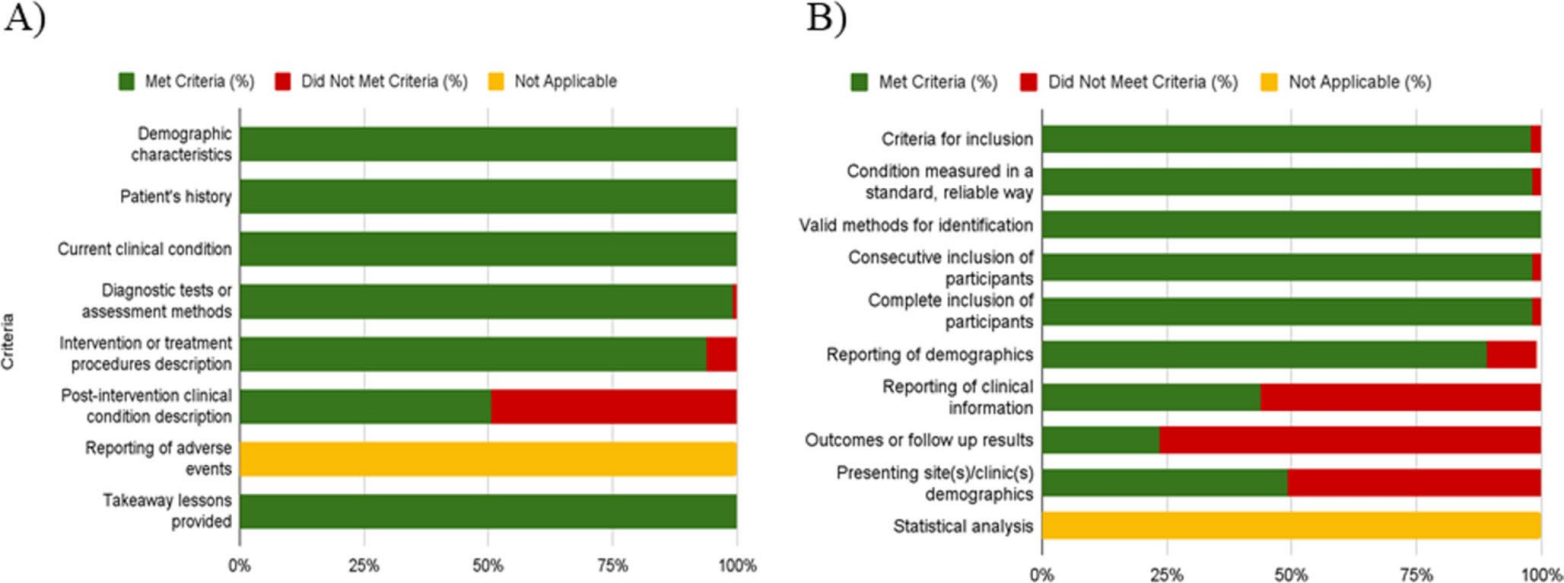
Risk of bias assessment according to the Joanna Briggs Institute (JBI) critical appraisal tool for case reports and case series in systematic reviews

Results of the risk of bias assessment are summarized in Fig. 4 and detailed in **Supplementary Tables **2** and **3. All case reports (*n* = 113/100.00%) clearly described the patient’s demographic characteristics, history, and current clinical condition at the time of presentation. Most studies (*n* = 112/99.11%) adequately reported the diagnostic tests or assessment methods and their results. The intervention or treatment procedures were clearly described in 106 studies (93.80%). However, only 57 articles (50.44%) provided a clear description of the post-intervention clinical condition. The reporting of adverse events or unanticipated outcomes was considered not applicable for all studies. Finally, takeaway lessons were provided in all included reports (*n* = 113/100.00%).

Regarding case series, most papers (*n* = 54/98.18%) clearly described the criteria for inclusion of participants and measured the condition in a standard, reliable way for all participants included in the case series. All studies (*n* = 55/100.00%) used valid methods for the identification of the condition for all participants included in the case series, and most case series had consecutive inclusion of participants (*n* = 54/98.18%) and complete inclusion of participants (*n* = 54/98.18%). The vast majority of studies (*n* = 49/89.09%) provided clear reporting of the demographics of the participants. However, most studies (*n* = 31/56.37%) did not provide clear reporting of the clinical information of the participants. Similarly, most studies (*n* = 42/76.37%) did not clearly report the outcomes or follow-up results of cases, and most studies (*n* = 28/50.91%) did not provide clear reporting of the presenting site(s)/clinic(s) demographic information. Finally, statistical analysis was considered not applicable for all papers. Overall, the methodological quality of the included evidence was heterogeneous, with incomplete reporting of clinical information, outcomes, and follow-up data being the most frequent limitations across studies. These patterns of bias were taken into account when interpreting the findings of this review, and studies with unclear or high-risk domains were interpreted with greater caution.

## Discussion

This systematic review synthesized data from 2,734 cases of NPDC, providing a comprehensive overview of their clinicopathological features. Demographically, NPDCs account for fewer than 5% of all jaw cysts [8], yet they represent up to 80% of non-odontogenic lesions (NOLs), making them the most frequently reported NOLs in retrospective studies [10–15]. Asia showed a higher relative prevalence of NPDC in this systematic review. However, this pattern should be interpreted cautiously, as it may reflect not only true epidemiological differences but also the disproportionate number of cases reported and published from that region. Differences in research output, diagnostic practices, and publication trends across continents may therefore inflate the apparent prevalence relative to other regions.

NPDC affects individuals across a wide age range, with peak occurrence in middle-aged adults, and it is also among the most reported NOLs in both younger [16] and older [17] patients. In the present review, a slight male predominance was observed, which is in accordance with previous reports [18]. Although the reasons for this sex-based distribution are not fully understood, some hypotheses suggest the influence of anatomical factors related to the nasopalatine canal. Thakur et al. (2013) reported statistically significant sex-based variations in canal length, with males exhibiting longer nasopalatine canals. This finding may be associated with the greater cranio-caudal dimension of the male face compared with the female face, suggesting that variations in nasopalatine canal size may reflect overall facial proportions [19].

Although NPDCs are often asymptomatic, a significant proportion of cases may present symptoms such as pain or drainage, especially when the lesion reaches larger dimensions or becomes secondarily infected [20]– [21]. These findings suggest that submucosal involvement and the lesion’s proximity to the palatal surface may play an important role in the clinical detection of a palatal prominence, since it is unlikely that a cyst developing in this region would cause extensive bone resorption without resulting in a noticeable palatal swelling [22]. Additionally, cases presenting both mucosal drainage and palatal swelling may difficult the diagnosis due to clinical overlap with endodontic pathologies, especially because both conditions manifest in the apical region of the anterior teeth [23]. This overlap highlights the importance of integrating clinical, radiographic, and pulp vitality assessments when evaluating anterior maxillary lesions, as misdiagnosis may lead to unnecessary endodontic treatment or delayed management of NPDC.

NPDC has been identified as one of the most common benign NOL that can mimic periapical pathology, accounting for 10.79% of such cases in a recent systematic review [24]. Our findings are consistent with this pattern, as the periapical cyst was the second most frequent initial diagnosis reported in the included cases, surpassed only by a preliminary suspicion of NPDC itself. Although diagnostic errors involving NPDC are not exceedingly common, the consistent presence of this lesion among the main differential diagnoses for anterior maxillary radiolucencies reinforces the importance of maintaining a broad diagnostic perspective and integrating clinical examination and imaging tools [25, 26]. A key distinguishing feature between NPDC and chronic periapical lesions is that the pulp of adjacent teeth usually remains vital even in large NPDCs, which is generally not observed in periapical chronic lesions arising from necrotic teeth [23]. However, 47 cases in the present study exhibited pulp necrosis in the involved teeth, suggesting a potential concomitant presentation of both conditions [27]. This finding underscores the need for careful diagnostic interpretation, as concurrent endodontic disease may obscure the radiographic and clinical hallmarks typically associated with NPDC.

The diagnostic approach to NPDCs relies heavily on complementary imaging methods. In our sample, the frequent use of periapical radiographs and CT scan, either alone or in combination, is consistent with current diagnostic routines in dentistry [28]. CT imaging, in particular, is a powerful tool for assessing intraosseous jaw lesions, especially in the maxilla, offering three-dimensional visualization without overlap of anatomical structures [29]. Notably, MRI was employed in only 3 cases, a limitation that may be attributed to its limited familiarity in dental settings. However, MRI offers a unique advantage by enabling the evaluation of lesion content, which could be particularly valuable in distinguishing a NPDC from endodontic pathologies [30, 31]. Emerging evidence also highlights the potential of dentistry-dedicated MRI systems, which are currently under validation [32]. These advances may pave the way for broader adoption of MRI in dental diagnostics, offering new perspectives for the evaluation of radiolucent lesions such as NPDC.

Classically, NPDCs appear radiographically as well-defined, unilocular radiolucent lesions [23], a pattern also observed in the present systematic review. Most NPDC are incidentally discovered during routine dental radiographic examinations, and typically appear as heart or pear-shaped radiolucencies, a feature often attributable to the superimposition of the anterior nasal spine [6, 8]. The literature describes NPDCs as typically measuring between 1 and 2 centimeters and located between the central incisors [6]. In the present systematic review, however, the mean lesion size was 2.59 cm, suggesting that the cases available in the published literature (especially case reports and case series) may disproportionately represent larger or more clinically evident cysts. This potential publication bias should be considered when interpreting lesion dimensions. NPDCs rarely cause root resorption or tooth displacement, and when such changes occur, they are typically associated with larger lesions [33]. Consistent with this association, 16 cases in the present study demonstrated root resorption, with a mean lesion size of 3.6 cm. This finding reinforces the established relationship between lesion size and its potential to exert pressure on adjacent structures.

Histopathological analysis showed that most frequently observed combinations were stratified squamous with pseudostratified ciliated columnar epithelium, followed by stratified squamous with pseudostratified ciliated columnar and simple cuboidal epithelium. In cases presenting a single epithelial type, stratified squamous was the most common, followed by pseudostratified ciliated columnar. According to the 5th Edition WHO Classification of Tumours, the diagnosis of NPDC relies on essential criteria, including an epicenter within the incisive canal and a lining composed of non-keratinized squamous or respiratory epithelium, as well as desirable criteria, such as the presence of neurovascular bundles within the cyst wall [8]. Cysts located in the superior portion are typically lined by respiratory epithelium, whereas those closer to the oral cavity exhibit stratified squamous epithelium [8]. This variability in epithelial lining may lead to diagnostic challenges, particularly for less experienced pathologists [1]. Notably, neurovascular bundles were identified in 95.82% of reported cases. The frequent presence of neurovascular bundles, glandular tissue, and even cartilage supports the developmental nature of the NPDC and aids in distinguishing it from inflammatory cysts [6]. These findings underscore the importance of correlating histopathology with radiographic and clinical features, as reliance on epithelial lining alone may lead to misdiagnosis, particularly in atypical presentations or when tissue sampling is limited.

Surgical enucleation was the preferred treatment approach, with recurrence rates reported to be extremely rare, as documented in the literature [6]. In cases of large cysts or challenging locations, marsupialization or decompression may be employed to reduce cyst pressure and size, thereby facilitating enucleation in a single piece [34]. Nevertheless, complete removal of the lesion remains essential to further minimize the risk of recurrence. Among the marsupialization cases included in this review, four were treated via transnasal endoscopic marsupialization, a technique considered less invasive than the conventional dental approach. This method allows preservation of most affected teeth and is described as a simple and effective procedure for maxillary cysts extending into the maxillary sinus or nasal floor, with lower risk, reduced trauma, and no impairment of nasal function [35, 36]. However, interpretation of treatment outcomes must be made cautiously, as follow-up information was inconsistently reported across studies, limiting the ability to derive reliable recurrence estimates. Although follow-up information was inconsistently reported, the available data suggest a favorable prognosis when complete removal of the lesion is achieved. Given the predominance of case reports and small case series in the available evidence, standardization of follow-up duration and documentation in future studies is therefore essential to more accurately determine long-term outcomes.

This systematic review has some limitations that should be acknowledged. First, the lack of detailed patient information in several studies, particularly case series, highlights the importance of using standardized reporting tools such as the CARE (CAse REports) and STROBE (Strengthening the Reporting of Observational Studies in Epidemiology) guidelines to ensure more comprehensive and uniform data reporting. Second, the possibility of publication bias cannot be ruled out, as unusual or symptomatic cases are more likely to be published than typical, asymptomatic presentations, potentially skewing the perceived clinical spectrum of NPDC. Third, the number of studies without full-text access represents an important limitation, especially because many were published in decades prior to the digitalization of scientific content, which makes it difficult to obtain complete data. The predominance of descriptive study designs also restricts the ability to draw population-level inferences, and incomplete or inconsistent follow-up reporting complicates the accurate estimation of recurrence rates and long-term outcomes. Despite these limitations, one of the main strengths of this review lies in the broad scope of the systematic review, which compiled a substantial number of cases, allowing for a thorough analysis of the clinical, radiographic, and histopathological features of NPDC. Future studies should prioritize standardized reporting, uniform diagnostic criteria, and prospective data collection with systematic long-term follow-up to enhance comparability, strengthen the quality of available evidence, and improve diagnostic confidence.

## Conclusion

In conclusion, NPDCs primarily affected adult males and typically presented as asymptomatic or slow-growing lesions. CT scan was frequently used to aid diagnosis. Histopathological analysis confirmed the presence of diverse epithelial linings and embryonic remnants, consistent with its developmental origin. Overall, NPDC showed an indolent clinical course with low recurrence, and outcomes are favorable when properly diagnosed and managed. Continued efforts toward standardized reporting and long-term follow-up are essential to strengthen the evidence base and improve diagnostic accuracy.

## Other information

### Protocol and registration

This systematic review was conducted according to the guidelines of the Preferred Reporting Items for Systematic Reviews and MetaAnalyses (PRISMA) Statement [37]. A protocol was drafted and registered in the International Prospective Register of Systematic Reviews (PROSPERO). The following number was assigned to the systematic review: CRD42024563227.

## Supplementary information

Below is the link to the electronic supplementary material.

Supplementary File 1 (DOCX 9.08 KB)

Supplementary File 2 (DOCX 20.1 KB)

Supplementary File 3 (DOCX 14.8 KB)

Supplementary File 4 (DOCX 21.4 KB)

Supplementary File 5 (DOCX 22.5 KB)

## Data Availability

No datasets were generated or analysed during the current study.
