# Supplementary material for: Clinicopathological features of the nasopalatine duct cyst: A systematic review
Source: Oral Maxillofac Surg. 2026 Feb 21;30(1):31. doi: 10.1007/s10006-026-01515-x (PMC12923490; doi:10.1007/s10006-026-01515-x)
Supplement: Supplementary file 1 — (DOCX 9.08 KB) [file 10006_2026_1515_MOESM1_ESM.docx]

**Supplementary Table 1.** Search strategies were performed for each bibliographic database.

| **Database** | **Search strategy**  (Search date: October 25^st^, 2025) | **Results** |
| --- | --- | --- |
| PubMed | "Nasopalatine duct cyst" OR "Incisive canal cyst" OR "Midline maxillary cyst" OR "Anterior median palatine cyst" OR "Incisive canal cyst" OR "Incisor duct cyst" OR "Nasopalatine canal cyst" OR "Median anterior maxillary cyst" OR "Anterior midline maxillary cyst" OR "Cyst of the nasopalatine foramen" OR "Cyst of the palatine papilla" | 364 |
| Scopus | TITLE-ABS-KEY("Nasopalatine duct cyst" OR "Incisive canal cyst" OR "Midline maxillary cyst" OR "Anterior median palatine cyst" OR "Incisive canal cyst" OR "Incisor duct cyst" OR "Nasopalatine canal cyst" OR "Median anterior maxillary cyst" OR "Anterior midline maxillary cyst" OR "Cyst of the nasopalatine foramen" OR "Cyst of the palatine papilla") | 235 |
| Embase | ("Nasopalatine duct cyst" OR "Incisive canal cyst" OR "Midline maxillary cyst" OR "Anterior median palatine cyst" OR "Incisive canal cyst" OR "Incisor duct cyst" OR "Nasopalatine canal cyst" OR "Median anterior maxillary cyst" OR "Anterior midline maxillary cyst" OR "Cyst of the nasopalatine foramen" OR "Cyst of the palatine papilla") | 179 |
| Web of Science | ("Nasopalatine duct cyst" OR "Incisive canal cyst" OR "Midline maxillary cyst" OR "Anterior median palatine cyst" OR "Incisive canal cyst" OR "Incisor duct cyst" OR "Nasopalatine canal cyst" OR "Median anterior maxillary cyst" OR "Anterior midline maxillary cyst" OR "Cyst of the nasopalatine foramen" OR "Cyst of the palatine papilla") (Topic) | 134 |
| Ovid | ("Nasopalatine duct cyst" OR "Incisive canal cyst" OR "Midline maxillary cyst" OR "Anterior median palatine cyst" OR "Incisive canal cyst" OR "Incisor duct cyst" OR "Nasopalatine canal cyst" OR "Median anterior maxillary cyst" OR "Anterior midline maxillary cyst" OR "Cyst of the nasopalatine foramen" OR "Cyst of the palatine papilla") | 21 |
| LILACS | ("Nasopalatine duct cyst" OR "Incisive canal cyst" OR "Midline maxillary cyst" OR "Anterior median palatine cyst" OR "Incisive canal cyst" OR "Incisor duct cyst" OR "Nasopalatine canal cyst" OR "Median anterior maxillary cyst" OR "Anterior midline maxillary cyst" OR "Cyst of the nasopalatine foramen" OR "Cyst of the palatine papilla" OR “Cisto do ducto nasopalatino” OR “Cisto do canal incisivo” OR “quiste del conducto nasopalatino” OR “quiste del canal incisivo”) | 43 |
| Google Scholar | First 150 more relevant hits. ("Nasopalatine duct cyst" OR "Incisive canal cyst" OR "Midline maxillary cyst" OR "Anterior median palatine cyst" OR "Incisive canal cyst" OR "Incisor duct cyst" OR "Nasopalatine canal cyst" OR "Median anterior maxillary cyst" OR "Anterior midline maxillary cyst" OR "Cyst of the nasopalatine foramen" OR "Cyst of the palatine papilla") | 150 |
| ProQuest | TI,AB("Nasopalatine duct cyst" OR "Incisive canal cyst" OR "Midline maxillary cyst" OR "Anterior median palatine cyst" OR "Incisive canal cyst" OR "Incisor duct cyst" OR "Nasopalatine canal cyst" OR "Median anterior maxillary cyst" OR "Anterior midline maxillary cyst" OR "Cyst of the nasopalatine foramen" OR "Cyst of the palatine papilla") | 49 |
