# Supplementary material for: Clinicopathological features of the nasopalatine duct cyst: A systematic review
Source: Oral Maxillofac Surg. 2026 Feb 21;30(1):31. doi: 10.1007/s10006-026-01515-x (PMC12923490; doi:10.1007/s10006-026-01515-x)
Supplement: Supplementary file 2 — (DOCX 20.1 KB) [file 10006_2026_1515_MOESM2_ESM.docx]

**Supplementary Table 2.** Results of the risk of bias assessment for the isolated studies according to the Joanna Briggs Institute critical appraisal tool for case reports.

| ***Author, Year*** | ***Were patient’s demographic characteristics clearly described?*** | ***Was the patient’s history clearly described and presented as a timeline?*** | ***Was the current clinical condition of the patient on presentation clearly described?*** | ***Were diagnostic tests or assessment methods and the results clearly described?*** | ***Was the intervention(s) or treatment procedure(s) clearly described?*** | ***Was the post-intervention clinical condition clearly described?*** | ***Were adverse events (harms) or unanticipated events identified and described?*** | ***Does the case report provide takeaway lessons?*** |
| --- | --- | --- | --- | --- | --- | --- | --- | --- |
| Abassi et al., 2015 | Yes | Yes | Yes | Yes | No | Yes | NA | Yes |
| Alassaf et al., 2023 | Yes | Yes | Yes | Yes | Yes | Yes | NA | Yes |
| Albayram et al., 2001 | Yes | Yes | Yes | Yes | Yes | No | NA | Yes |
| Alhozgi et al., 2019 | Yes | Yes | Yes | Yes | Yes | Yes | NA | Yes |
| Almeida et al., 2021 | Yes | Yes | Yes | No | Yes | Yes | NA | Yes |
| Álvarez, 1995 | Yes | Yes | Yes | Yes | Yes | No | NA | Yes |
| Aparna et al., 2014 | Yes | Yes | Yes | Yes | Yes | Yes | NA | Yes |
| Ardakani et al., 2023 | Yes | Yes | Yes | Yes | Yes | Yes | NA | Yes |
| Austin, 1947 | Yes | Yes | Yes | Yes | Yes | No | NA | Yes |
| Auteri et al., 2017 | Yes | Yes | Yes | Yes | Yes | No | NA | Yes |
| Baby et al., 2018 | Yes | Yes | Yes | Yes | Yes | No | NA | Yes |
| Borgonovo et al., 2011 | Yes | Yes | Yes | Yes | Yes | Yes | NA | Yes |
| Buschini et al., 2015 | Yes | Yes | Yes | Yes | Yes | Yes | NA | Yes |
| Casado et al., 2008 | Yes | Yes | Yes | Yes | Yes | No | NA | Yes |
| Cerine et al., 1967 | Yes | Yes | Yes | Yes | Yes | No | NA | Yes |
| Çetin et al., 2012 | Yes | Yes | Yes | Yes | Yes | Yes | NA | Yes |
| Chen et al., 2011 | Yes | Yes | Yes | Yes | Yes | Yes | NA | Yes |
| Cicciù et al., 2010 | Yes | Yes | Yes | Yes | Yes | Yes | NA | Yes |
| Ciola & Catena, 1972 | Yes | Yes | Yes | Yes | Yes | Yes | NA | Yes |
| Cohen, 1941 | Yes | Yes | Yes | Yes | Yes | No | NA | Yes |
| Concha et al., 2023 | Yes | Yes | Yes | Yes | Yes | No | NA | Yes |
| Coz-Fano et al., 2014 | Yes | Yes | Yes | Yes | Yes | Yes | NA | Yes |
| Curtin et al., 1984 | Yes | Yes | Yes | Yes | Yes | No | NA | Yes |
| Dantas et al., 2014 | Yes | Yes | Yes | Yes | Yes | Yes | NA | Yes |
| Dedhia et al., 2013 | Yes | Yes | Yes | Yes | Yes | No | NA | Yes |
| Deshpande et al., 2025 | Yes | Yes | Yes | Yes | Yes | Yes | NA | Yes |
| Doherty, 1940 | Yes | Yes | Yes | Yes | Yes | No | NA | Yes |
| Ely et al., 2001 | Yes | Yes | Yes | Yes | Yes | No | NA | Yes |
| Farroco et al., 2022 | Yes | Yes | Yes | Yes | Yes | Yes | NA | Yes |
| Fujoka-Kobayashi et al., 2023 | Yes | Yes | Yes | Yes | Yes | Yes | NA | Yes |
| Gadicherla et al., 2024 | Yes | Yes | Yes | Yes | Yes | No | NA | Yes |
| Gawande et al., 2012 | Yes | Yes | Yes | Yes | Yes | No | NA | Yes |
| Gingell et al., 1985 | Yes | Yes | Yes | Yes | Yes | No | NA | Yes |
| Gnanasekhar et al., 1995 | Yes | Yes | Yes | Yes | Yes | Yes | NA | Yes |
| Gopal et al., 2015 | Yes | Yes | Yes | Yes | Yes | No | NA | Yes |
| Gowrishankar et al., 2023 | Yes | Yes | Yes | Yes | Yes | Yes | NA | Yes |
| Gulabivala & Briggs, 1992 | Yes | Yes | Yes | Yes | Yes | No | NA | Yes |
| Harris & Brown, 1997 | Yes | Yes | Yes | Yes | Yes | No | NA | Yes |
| Hasan et al., 2022 | Yes | Yes | Yes | Yes | Yes | Yes | NA | Yes |
| Hegde & Shetty, 2006 | Yes | Yes | Yes | Yes | Yes | No | NA | Yes |
| Hilfer et al., 2013 | Yes | Yes | Yes | Yes | Yes | No | NA | Yes |
| Igreja et al., 2005 | Yes | Yes | Yes | Yes | Yes | Yes | NA | Yes |
| Jan et al., 2023 | Yes | Yes | Yes | Yes | Yes | No | NA | Yes |
| Kagoya et al., 2022 | Yes | Yes | No | Yes | Yes | No | NA | Yes |
| Kang et al., 2020 | Yes | Yes | Yes | Yes | No | No | NA | Yes |
| Kawata et al., 2000 | Yes | Yes | Yes | Yes | Yes | Yes | NA | Yes |
| Kerkar et al., 2015 | Yes | Yes | Yes | Yes | No | No | NA | Yes |
| Khalil & Albash, 2024 | Yes | Yes | Yes | Yes | Yes | No | NA | Yes |
| Khan et al., 2024 | Yes | Yes | Yes | Yes | Yes | Yes | NA | Yes |
| Kim et al., 2012 | Yes | Yes | Yes | Yes | No | Yes | NA | Yes |
| Kim et al., 2023 | Yes | Yes | Yes | Yes | Yes | Yes | NA | Yes |
| Kobashi et al., 2017 | Yes | Yes | Yes | Yes | Yes | No | NA | Yes |
| Kobayashi et al., 2022 | Yes | Yes | Yes | Yes | Yes | Yes | NA | Yes |
| Levy et al., 2021 | Yes | Yes | Yes | Yes | Yes | Yes | NA | Yes |
| Mahajan et al., 2021 | Yes | Yes | Yes | Yes | No | No | NA | Yes |
| Makihara et al., 2016 | Yes | Yes | Yes | Yes | Yes | Yes | NA | Yes |
| Martins et al., 2007 | Yes | Yes | Yes | Yes | Yes | Yes | NA | Yes |
| Matijević et al., 2007 | Yes | Yes | Yes | Yes | Yes | No | NA | Yes |
| McCrea, 2014 | Yes | Yes | Yes | Yes | Yes | Yes | NA | Yes |
| Mesquita et al., 2014 | Yes | Yes | Yes | Yes | Yes | No | NA | Yes |
| Mullaney & Lenox, 1970 | Yes | Yes | Yes | Yes | Yes | Yes | NA | Yes |
| Nelson & Linfesty, 2010 | Yes | Yes | Yes | Yes | Yes | No | NA | Yes |
| Noleto et al., 2010 | Yes | Yes | Yes | Yes | Yes | Yes | NA | Yes |
| Öçbe et al., 2024 | Yes | Yes | Yes | Yes | Yes | No | NA | Yes |
| Ohno et al., 2003 | Yes | Yes | Yes | Yes | Yes | Yes | NA | Yes |
| Ohtsuka et al., 2021 | Yes | Yes | Yes | Yes | Yes | Yes | NA | Yes |
| Oliva et al., 2020 | Yes | Yes | Yes | Yes | Yes | Yes | NA | Yes |
| Oliveira et al., 2009 | Yes | Yes | Yes | Yes | Yes | Yes | NA | Yes |
| Oliveira et al., 2024 | Yes | Yes | Yes | Yes | Yes | Yes | NA | Yes |
| Oliver et al., 1992 | Yes | Yes | Yes | Yes | Yes | No | NA | Yes |
| Panjwani et al., 2014 | Yes | Yes | Yes | Yes | Yes | Yes | NA | Yes |
| Perez et al., 2022 | Yes | Yes | Yes | Yes | Yes | No | NA | Yes |
| Perumal, 2013 | Yes | Yes | Yes | Yes | Yes | No | NA | Yes |
| Pontes et al., 2014 | Yes | Yes | Yes | Yes | No | No | NA | Yes |
| Popli et al., 2022 | Yes | Yes | Yes | Yes | Yes | No | NA | Yes |
| Queiroz et al., 2011 | Yes | Yes | Yes | Yes | Yes | Yes | NA | Yes |
| Rangaswamy et al., 2018 | Yes | Yes | Yes | Yes | Yes | Yes | NA | Yes |
| Redman, 1974 | Yes | Yes | Yes | Yes | Yes | No | NA | Yes |
| Rojas et al., 2014 | Yes | Yes | Yes | Yes | No | No | NA | Yes |
| Sadegghi & Angell, 1985 | Yes | Yes | Yes | Yes | Yes | No | NA | Yes |
| Salamm et al., 2011 | Yes | Yes | Yes | Yes | Yes | No | NA | Yes |
| Salgado et al., 2014 | Yes | Yes | Yes | Yes | Yes | Yes | NA | Yes |
| Sane et al., 2014 | Yes | Yes | Yes | Yes | Yes | Yes | NA | Yes |
| Sankar et al., 2016 | Yes | Yes | Yes | Yes | Yes | No | NA | Yes |
| Saunders et al., 1968 | Yes | Yes | Yes | Yes | Yes | Yes | NA | Yes |
| Schiff et al., 1969 | Yes | Yes | Yes | Yes | Yes | No | NA | Yes |
| Schott et al., 1985 | Yes | Yes | Yes | Yes | Yes | No | NA | Yes |
| Scolozzi et al., 2008 | Yes | Yes | Yes | Yes | Yes | Yes | NA | Yes |
| Sheikh et al., 2024 | Yes | Yes | Yes | Yes | Yes | No | NA | Yes |
| Shilpa et al., 2012 | Yes | Yes | Yes | Yes | Yes | No | NA | Yes |
| Shinde et al., 2021 | Yes | Yes | Yes | Yes | Yes | Yes | NA | Yes |
| Shylaja et al., 2013 | Yes | Yes | Yes | Yes | Yes | No | NA | Yes |
| Spinelli et al., 1994 | Yes | Yes | Yes | Yes | Yes | No | NA | Yes |
| Srikanth et al., 2024 | Yes | Yes | Yes | Yes | Yes | No | NA | Yes |
| Srivastava et al., 2013 | Yes | Yes | Yes | Yes | Yes | No | NA | Yes |
| Stafne et al., 1947 | Yes | Yes | Yes | Yes | Yes | Yes | NA | Yes |
| Staretz et al., 1990 | Yes | Yes | Yes | Yes | Yes | No | NA | Yes |
| Sukegawa et al., 2015 | Yes | Yes | Yes | Yes | Yes | Yes | NA | Yes |
| Supreeda et al., 2025 | Yes | Yes | Yes | Yes | Yes | No | NA | Yes |
| Takasu et al., 2025 | Yes | Yes | Yes | Yes | Yes | Yes | NA | Yes |
| Takeshita et al., 2013 | Yes | Yes | Yes | Yes | Yes | Yes | NA | Yes |
| Tam, 1953 | Yes | Yes | Yes | Yes | Yes | Yes | NA | Yes |
| Tanaka et al., 2008 | Yes | Yes | No | Yes | Yes | Yes | NA | Yes |
| Terry & Bolanos, 1989 | Yes | Yes | Yes | Yes | Yes | Yes | NA | Yes |
| Thoma, 1943 | Yes | Yes | Yes | Yes | Yes | No | NA | Yes |
| Torres et al., 2008 | Yes | Yes | Yes | Yes | Yes | Yes | NA | Yes |
| Trento et al., 2009 | Yes | Yes | Yes | Yes | Yes | Yes | NA | Yes |
| Yeom et al., 2021 | Yes | Yes | Yes | Yes | Yes | No | NA | Yes |
| Yogesh et al., 2014 | Yes | Yes | Yes | Yes | Yes | No | NA | Yes |
| Yokoyama & Suzaki, 1989 | Yes | Yes | Yes | Yes | Yes | Yes | NA | Yes |
| Yusa et al., 2025 | Yes | Yes | Yes | Yes | Yes | Yes | NA | Yes |
| Wu et al., 2013 | Yes | Yes | Yes | Yes | Yes | Yes | NA | Yes |
| **Total of Yes (%)** | **100.00** | **100.00** | **100.00** | **99.11** | **93.80** | **50.44** | **-** | **100.00** |

NA: Not applicable
