# Supplementary material for: Clinicopathological features of the nasopalatine duct cyst: A systematic review
Source: Oral Maxillofac Surg. 2026 Feb 21;30(1):31. doi: 10.1007/s10006-026-01515-x (PMC12923490; doi:10.1007/s10006-026-01515-x)
Supplement: Supplementary file 3 — (DOCX 14.8 KB) [file 10006_2026_1515_MOESM3_ESM.docx]

**Supplementary Table 3.** Results of the risk of bias assessment for the isolated studies according to the Joanna Briggs Institute critical appraisal tool for series of cases.

| ***Author, Year*** | ***Were there clear criteria for inclusion in the case series?*** | ***Was the condition measured in a standard, reliable way for all participants included in the case series?*** | ***Were valid methods used for identification of the condition for all participants included in the case series?*** | ***Did the case series have consecutive inclusion of participants?*** | ***Did the case series have complete inclusion of participants?*** | ***Was there clear reporting of the demographics of the participants in the study?*** | ***Was there clear reporting of clinical information of the participants?*** | ***Were the outcomes or follow up results of cases clearly reported?*** | ***Was there clear reporting of the presenting site(s)/clinic(s) demographic information?*** | ***Was statistical analysis appropriate?*** |
| --- | --- | --- | --- | --- | --- | --- | --- | --- | --- | --- |
| Açikgöz et al., 2012 | Yes | Yes | Yes | Yes | Yes | Yes | Yes | No | Yes | NA |
| Aldelaimi & Khalil, 2012 | No | No | Yes | Yes | Yes | Yes | Yes | No | Yes | NA |
| Allard et al., 1981 | Yes | Yes | Yes | Yes | Yes | Yes | Yes | Yes | Yes | NA |
| Alqaied, 2012 | Yes | Yes | Yes | Yes | Yes | Yes | No | No | Yes | NA |
| Anneroth et al., 1986 | Yes | Yes | Yes | Yes | Yes | Yes | Yes | Yes | Yes | NA |
| Bachur et al., 2009 | Yes | Yes | Yes | Yes | Yes | Yes | Yes | No | Yes | NA |
| Barros et al., 2018 | Yes | Yes | Yes | Yes | Yes | Yes | No | No | No | NA |
| Bayrakdar et al., 2020 | Yes | Yes | Yes | Yes | Yes | No | No | No | No | NA |
| Becconsall-Ryan et al., 2011 | Yes | Yes | Yes | Yes | Yes | Yes | No | No | No | NA |
| Bodin et al., 1986 | Yes | Yes | Yes | Yes | Yes | Yes | Yes | Yes | Yes | NA |
| Butt et al., 2011 | Yes | Yes | Yes | Yes | Yes | No | No | No | No | NA |
| Cavalcante et al., 2021 | Yes | Yes | Yes | Yes | Yes | Yes | Yes | Yes | Yes | NA |
| Cechetti et al., 2012 | Yes | Yes | Yes | Yes | Yes | Yes | No | No | No | NA |
| Danuthai et al., 2024 | Yes | Yes | Yes | Yes | Yes | Yes | No | No | No | NA |
| Elliot et al., 2004 | Yes | Yes | Yes | Yes | Yes | Yes | Yes | Yes | Yes | NA |
| Faitaroni et al., 2011 | Yes | Yes | Yes | Yes | Yes | Yes | Yes | No | Yes | NA |
| Fialho et al., 2017 | Yes | Yes | Yes | Yes | Yes | Yes | Yes | No | No | NA |
| Francoli et al., 2008 | Yes | Yes | Yes | Yes | Yes | Yes | Yes | Yes | Yes | NA |
| Franklin et al., 2021 | Yes | Yes | Yes | Yes | Yes | Yes | No | No | No | NA |
| Grossmann et al., 2007 | Yes | Yes | Yes | Yes | Yes | No | No | No | No | NA |
| Hedin et al., 1978 | Yes | Yes | Yes | Yes | Yes | Yes | No | Yes | No | NA |
| Hertzanu et al., 1985 | Yes | Yes | Yes | Yes | Yes | Yes | Yes | No | Yes | NA |
| Hosgor et al., 2019 | Yes | Yes | Yes | Yes | Yes | Yes | No | No | Yes | NA |
| Hyde, 1938 | Yes | Yes | Yes | No | No | Yes | No | No | No | NA |
| Ito et al., 2016 | Yes | Yes | Yes | Yes | Yes | Yes | No | No | No | NA |
| Kang et al., 2014 | Yes | Yes | Yes | Yes | Yes | Yes | No | No | No | NA |
| Kilinc et al., 2017 | Yes | Yes | Yes | Yes | Yes | Yes | No | No | No | NA |
| Kosanwat et al., 2021 | Yes | Yes | Yes | Yes | Yes | Yes | No | No | No | NA |
| Kreidler et al., 1993 | Yes | Yes | Yes | Yes | Yes | No | No | No | No | NA |
| Krongbaramee et al., 2023 | Yes | Yes | Yes | Yes | Yes | No | No | No | No | NA |
| Mealey et al., 1993 | Yes | Yes | Yes | Yes | Yes | Yes | Yes | No | Yes | NA |
| Mittal & Mohandas et al., 2017 | Yes | Yes | Yes | Yes | Yes | Yes | Yes | No | Yes | NA |
| Naini et al., 2017 | Yes | Yes | Yes | Yes | Yes | Yes | No | No | No | NA |
| Nonaka et al., 2011 | Yes | Yes | Yes | Yes | Yes | Yes | No | No | No | NA |
| Nortjé & Farman, 1978 | Yes | Yes | Yes | Yes | Yes | Yes | Yes | No | No | NA |
| Nortjé & Wood, 1988 | Yes | Yes | Yes | Yes | Yes | Yes | No | No | No | NA |
| Oliveira et al., 2017 | Yes | Yes | Yes | Yes | Yes | Yes | Yes | Yes | Yes | NA |
| Ortega et al., 2007 | Yes | Yes | Yes | Yes | Yes | Yes | No | No | Yes | NA |
| Park et al., 2012 | Yes | Yes | Yes | Yes | Yes | Yes | Yes | Yes | Yes | NA |
| Ramos Peña et al., 2025 | Yes | Yes | Yes | Yes | Yes | Yes | Yes | No | Yes | NA |
| Rees et al., 2025 | Yes | Yes | Yes | Yes | Yes | Yes | No | No | No | NA |
| Righini et al., 2004 | Yes | Yes | Yes | Yes | Yes | Yes | No | No | No | NA |
| Sarangi et al., 2024 | Yes | Yes | Yes | Yes | Yes | Yes | Yes | Yes | Yes | NA |
| Sebastian et al., 2025 | Yes | Yes | Yes | Yes | Yes | Yes | Yes | Yes | Yes | NA |
| Sun & Dym, 2023 | Yes | Yes | Yes | Yes | Yes | Yes | Yes | Yes | Yes | NA |
| Suter et al., 2011 (a) | Yes | Yes | Yes | Yes | Yes | Yes | Yes | No | Yes | NA |
| Suter et al., 2011 (b) | Yes | Yes | Yes | Yes | Yes | Yes | No | No | No | NA |
| Suter et al., 2015 | Yes | Yes | Yes | Yes | Yes | Yes | No | No | No | NA |
| Swanson et al., 1991 | Yes | Yes | Yes | Yes | Yes | Yes | No | No | No | NA |
| Syebele et al., 2018 | Yes | Yes | Yes | Yes | Yes | No | No | No | No | NA |
| Tamiolakis et al., 2019 | Yes | Yes | Yes | Yes | Yes | Yes | No | No | Yes | NA |
| Tsuneki et al., 2013 | Yes | Yes | Yes | Yes | Yes | Yes | No | No | No | NA |
| Uchoa-Vasconcelos et al., 2014 | Yes | Yes | Yes | Yes | Yes | Yes | No | No | No | NA |
| Ueda et al., 2019 | Yes | Yes | Yes | Yes | Yes | Yes | No | No | No | NA |
| Vasconcelos et al., 1999 | Yes | Yes | Yes | Yes | Yes | Yes | Yes | Yes | Yes | NA |
|  | Yes | Yes | Yes | Yes | Yes | Yes | No | No | No | NA |
| **Total of Yes (%)** | **98.18** | **98.18** | **100.00** | **98.18** | **98.18** | **89.09** | **43.63** | **23.63** | **49.09** | **-** |

NA: Not applicable
