# Supplementary material for: Clinicopathological features of the nasopalatine duct cyst: A systematic review
Source: Oral Maxillofac Surg. 2026 Feb 21;30(1):31. doi: 10.1007/s10006-026-01515-x (PMC12923490; doi:10.1007/s10006-026-01515-x)
Supplement: Supplementary file 4 — (DOCX 21.4 KB) [file 10006_2026_1515_MOESM4_ESM.docx]

**Supplementary Table 4.** Excluded articles and reasons for exclusion.

|  | **References** | **Reasons for exclusion** |
| --- | --- | --- |
| **1.** | Alassaf MS, Abu Aof MM, Othman A, Zaki H, Almutairi AG. Coexistence of a Nasopalatine Duct Cyst and Radicular Cyst: A Unique Clinical Presentation. Cureus. 2023;15(10): e46774. https://doi.org/10.7759/cureus.46774. | 3 |
| **2.** | Albayram MS, Sciubba J, Zinreich SJ. Radiology Quiz Case. Arch Otolaryngol Head Neck Surg. 2001;127:1283-5. | 3 |
| **3.** | Allard RH. Proefschriften 25 jaar na dato 8. Niet-dentogene cysten in het hoofd-hals-gebied [Dissertations 25 years after date 8. Non-odontogenic cysts of the oral regions]. Ned Tijdschr Tandheelkd. 2006;113(7):278-83. | 3 |
| **4.** | Al-Shamiri HM, Elfaki S, Al-Maweri SA, Alaizari NA, Tarakji B. Development of Nasopalatine Duct Cyst in Relation to Dental Implant Placement. N Am J Med Sci. 2016;8(1):13-6. https://doi.org/10.4103/1947-2714.175187. | 2 |
| **5.** | Anneroth G, Hall G, Stuge U. Nasopalatine duct cyst. Int J Oral Maxillofac Surg. 1986;15(5):572-80. https://doi.org/10.1016/s0300-9785(86)80061-8. | 3 |
| **6.** | Aparna M, Chakravarthy A, Acharya SR, Radhakrishnan R. BMJ Case Rep. 2014:bcr2013200329. https://doi.org/10.1136/bcr-2013-200329. | 3 |
| **7.** | Bacci C. Is this a real "large median palatine cyst"? J Craniofac Surg. 2013;24(2):689-90. https://doi.org/10.1097/SCS.0b013e31827c85d5. | 2 |
| **8.** | Bahşi I, Orhan M, Kervancıoğlu P, Yalçın ED, Aktan AM. Anatomical evaluation of nasopalatine canal on cone beam computed tomography images. Folia Morphol (Warsz). 2019;78(1):153-162. https://doi.org/10.5603/FM.a2018.0062. | 3 |
| **9.** | Bains SK, Bhatia A, Sodhi SS, Sharma A. Assessment of the Nasopalatine Canal in Patients Requiring Dental Implants in the Maxillary Anterior Region Using Cone Beam Computed Tomography. Cureus. 2023;15(12):e50643. https://doi.org/10.7759/cureus.50643. | 3 |
| **10.** | Barros CCDS, Santos HBP, Cavalcante IL, Rolim LSA, Pinto LP, de Souza LB. Clinical and histopathological features of nasopalatine duct cyst: A 47-year retrospective study and review of current concepts. J Craniomaxillofac Surg. 2018;46(2):264-268. https://doi.org/10.1016/j.jcms.2017.11.014. | 3 |
| **11.** | Basso ECB, de Paula Neto ER, Dib LL, Costa C. An unusual case of nasopalatine cyst in Brazilian population (Um caso incomum de cisto nasopalatino na população brasileira). J Health Sci Inst. 2012;30(3):292-4. | 1 |
| **12.** | Ben Slama L, Zoghbani A, Hidaya S. Kyste du canal nasopalatin. Rev Stomatol Chirur Maxillo-faciale. 2009;110(5):284-6. | 2 |
| **13.** | Boo-Chai K, Nortje CJ, Forman AG. Nasopalatine duct cyst. Plast Reconstr Surg. 1979;63(5):754. | 2 |
| **14.** | Brown FH, Houston GD, Lubow RM, Sagan MA. Cyst of the incisive (palatine) papilla. Report of a case. J Periodontol. 1987;58(4):274-5. https://doi.org/10.1902/jop.1987.58.4.274. | 1 |
| **15.** | Cam K, Sumer T, Zengin AZ. Radiographic evaluation of nasopalatine canal and its relationship with infraorbital foramen and foramen palatinum majus. J Stomatol Oral Maxillofac Surg. 2025;126(5S):102492. https://doi.org/10.1016/j.jormas.2025.102492. | 1 |
| **16.** | Carini F, Porcaro G, Monai D, Barbano L, Bucalo C, Pirrone F. Approccio terapeutico al paziente in terapia con bisfosfonati orali e affetto da cisti naso-palatina: caso clinico. Ital Oral Surg. 2012;11(5):226-33. | 1 |
| **17.** | Casado PL, Donner M, Pascarelli B, Derocy C, Duarte ME, Barboza EP. Immediate dental implant failure associated with nasopalatine duct cyst. Implant Dent. 2008;17(2):169-75. https://doi.org/10.1097/ID.0b013e3181776c52. | 3 |
| **18.** | Chen IJ, Lai PT, Jin YT, Chiang CP. Nasopalatine duct cyst - Diagnosis from the content in the cystic wall. J Dent Sci. 2023;18(3):1449-51. https://doi.org/10.1016/j.jds.2023.05.012. | 2 |
| **19.** | Çetin Ö, Tekkeşin MS, Tuskan A, Tuskan, AC. Nasopalatin kanal kisti (bir olgu sunumu). Journal of Istanbul University Faculty of Dentistry. 2012;46(1):59-64. | 3 |
| **20.** | Daley TD, Wysocki GP, Pringle GA. Relative incidence of odontogenic tumors and oral and jaw cysts in a Canadian population. Oral Surg Oral Med Oral Pathol. 1994;77(3):276-80. https://doi.org/10.1016/0030-4220(94)90299-2. | 1 |
| **21.** | Damm DD, Lu RJ, Rhoton RC. Concurrent nasopalatine duct cyst and bilateral mesiodens. Oral Surg Oral Med Oral Pathol. 1988;65(2):264-5. https://doi.org/10.1016/0030-4220(88)90179-x. | 2 |
| **22.** | Dedhia P, Dedhia S, Dhokar A, Desai A. Nasopalatine duct cyst. Case Rep Dent. 2013;2013:869516. https://doi.org/10.1155/2013/869516. | 3 |
| **23.** | El-Bardaie A, Nikai H, Takata T. Pigmented nasopalatine duct cyst. Report of 2 cases. Int J Oral Maxillofac Surg. 1989;18(3):138-9. https://doi.org/10.1016/s0901-5027(89)80109-2. | 2 |
| **24.** | Elo JA, Sun HB, Kang SY. Median maxillary alveolar osteolytic lesion in a 50-year-old female. Oral Surg Oral Med Oral Pathol Oral Radiol. 2017;123(1):3-7. https://doi.org/10.1016/j.oooo.2016.04.001. | 1 |
| **25.** | Ely N, Sheehy EC, McDonald F. Nasopalatine duct cyst: a case report. Int J Paediatr Dent. 2001;11:135-7. | 3 |
| **26.** | Erbaşar GN, Peker E, Barış E. Keratocystic odontogenic tumor simulating lateral periodontal cyst and nasopalatine duct cyst: Report of cases and literature review. Int J Clin Exp Med. 2016;9(9):18583-18592. | 1 |
| **27.** | Erkan AN, Yilmazer C, Yilmaz I, Bolat FA. Nasoalveolar cysts: review of 3 cases. ORL J Otorhinolaryngol Relat Spec. 2005;67(4):196-8. https://doi.org/10.1159/000086664. | 1 |
| **28.** | Escoda Francolí J, Almendros Marqués N, Berini Aytés L, Gay Escoda C. Nasopalatine duct cyst: report of 22 cases and review of the literature. Med Oral Patol Oral Cir Bucal. 2008;13(7):E438-43. https://www.medicinaoral.com/medoralfree01/v13i7/medoralv13i7p438.pdf | 3 |
| **29.** | Faitaroni LA, Bueno MR, Carvalhosa AA, Mendonça EF, Estrela C. Differential diagnosis of apical periodontitis and nasopalatine duct cyst. J Endod. 2011;37(3):403-10. https://doi.org/10.1016/j.joen.2010.11.022. | 3 |
| **30.** | Farzinnia G, Sasannia M, Torabi S, Rezazadeh F, Ranjbaran A, Azad A. Correlation between Clinical and Histopathological Diagnoses in Oral Cavity Lesions: A 12-Year Retrospective Study. Int J Dent. 2022;2022:1016495. https://doi.org/10.1155/2022/1016495. | 2 |
| **31.** | Gao X, Dai S, Yan X, Jia X, Huang B, Zhang H, Wei D, Guan X, Shi D, Meng H. Successful Treatment of Nasopalatine Duct Cyst After Maxillary Anterior Implant Surgery: A Case Report. J Oral Implantol. 2024;50(5):492-8. https://doi.org/10.1563/aaid-joi-D-23-00052. | 3 |
| **32.** | Gnanasekhar JD, Walvekar SV, al-Kandari AM, al-Duwairi Y. Misdiagnosis and mismanagement of a nasopalatine duct cyst and its corrective therapy. A case report. Oral Surg Oral Med Oral Pathol Oral Radiol Endod. 1995;80(4):465-70. https://doi.org/10.1016/s1079-2104(05)80372-5. | 3 |
| **33.** | Hertzanu Y, Cohen M, Mendelsohn DB. Nasopalatine duct cyst. Clin Radiol. 1985;36(2):153-8. https://doi.org/10.1016/s0009-9260(85)80099-4. | 3 |
| **34.** | Hilfer PB, Bergeron BE, Ozgul ES, Wong DK. Misdiagnosis of a nasopalatine duct cyst: a case report. J Endod. 2013;39(9):1185-8. https://doi.org/10.1016/j.joen.2013.04.033. | 3 |
| **35.** | Hisatomi M, Asaumi JI, Konouchi H, Matsuzaki H, Kishi K. MR imaging of nasopalatine duct cysts. Europ J Radiol. 2001;39(2):73-6. | 2 |
| **36.** | Honkura Y, Nomura K, Oshima H, Takata Y, Hidaka H, Katori Y. Bilateral endoscopic endonasal marsupialization of nasopalatine duct cyst. Clin Pract. 2015;5(1):748. https://doi.org/10.4081/cp.2015.748. | 1 |
| **37.** | Husain AAH, Schönegg D, Valdec S, Stadlinger B, Piccirelli M, Winklhofer S. Appearance of nasopalatine duct cysts on dental magnetic resonance imaging using a mandibular coil: Two case reports with a literature review. Imaging Sci Dent. 2023 Jun;53(2):161-168. https://doi.org/10.5624/isd.20220215. | 1 |
| **38.** | Ikeda H, Takata N, Kise Y, Ebata K, Mori M, Kuwada C, Nishiyama M, Iwase Y, Ninagawa Y, Naitoh M, Ariji E. Spread asymmetry to differentiate nasopalatine duct cysts from radicular cysts arising in the anterior maxilla on computed tomographic images. Oral Radiol. 2024;40(4):501-7. https://doi.org/10.1007/s11282-024-00761-7. | 2 |
| **39.** | Ito K, Hirahara N, Muraoka H, Sawada E, Tokunaga S, Komatsu T, Kaneda T. Graphical user interface-based convolutional neural network models for detecting nasopalatine duct cysts using panoramic radiography. Sci Rep. 2024;14(1):7699. https://doi.org/10.1038/s41598-024-57632-8. | 2 |
| **40.** | Janovic A, Bracanovic D, Antic S, Markovic-Vasiljkovic B. Morphological CBCT parameters for an accurate differentiation between nasopalatine duct cyst and the normal nasopalatine canal. Head Face Med. 2024;20(1):54. https://doi.org/10.1186/s13005-024-00458-6. | 3 |
| **41.** | Jones RS, Dillon J. Nonodontogenic Cysts of the Jaws and Treatment in the Pediatric Population. Oral Maxillofac Surg Clin North Am. 2016;28(1):31-44. https://doi.org/10.1016/j.coms.2015.08.001. | 2 |
| **42.** | Kerr JT, Steger J, Sorensen D. Midline maxillary odontogenic keratocyst. Ann Otol Rhinol Laryngol. 2004;113(9):688-90. https://doi.org/10.1177/000348940411300902. | 1 |
| **43.** | Kim DH, Jeon EJ, Park YS. Transnasal Endoscopic Microdebrider-Assisted Marsupialization of a Nasolabial Cyst and a Nasopalatine Duct Cyst. Otolaryngol Head Neck Surg. 2011;145(2_suppl):P268. | 2 |
| **44.** | Kim J, Nam IC, Hyun Yun S, Cho JH. A huge midline premaxillary cyst as a late complication of maxillary surgery. J Craniofac Surg. 2011;22(5):1903-5. https://doi.org/10.1097/SCS.0b013e31822ea676. | 2 |
| **45.** | Kim SJ, Moon JW, Lee HM. Huge Nasopalatine Duct Cyst Treated by Transnasal Endoscopic Marsupialization: A Case Report and Literature Review. Ear Nose Throat J. 2023;8:1455613231177986. https://doi.org/10.1177/01455613231177986. | 3 |
| **46.** | Kise Y, Kuwada C, Mori M, Fukuda M, Ariji Y, Ariji E. Deep learning system for distinguishing between nasopalatine duct cysts and radicular cysts arising in the midline region of the anterior maxilla on panoramic radiographs. Imaging Sci Dent. 2024;54(1):33-41. https://doi.org/10.5624/isd.20230169. | 2 |
| **47.** | Lang MJ, Lee YP, Hwang MJ, Chiang CP. Nasopalatine duct cyst - Case report. J Dent Sci. 2021;16(3):1047-9. https://doi.org/10.1016/j.jds.2021.03.004. | 2 |
| **48.** | Larmie J, Mandel L. Nasopalatine duct cyst. N Y State Dent J. 2021;87(5):32-5. | 2 |
| **49.** | Lee HS, Yang S, Han JY, Kang JH, Kim JE, Huh KH, Yi WJ, Heo MS, Lee SS. Automatic detection and classification of nasopalatine duct cyst and periapical cyst on panoramic radiographs using deep convolutional neural networks. Oral Surg Oral Med Oral Pathol Oral Radiol. 2024;138(1):184-95. https://doi.org/10.1016/j.oooo.2023.09.012. | 2 |
| **50.** | McCrea SJ. Nasopalatine duct cyst, a delayed complication to successful dental implant placement: diagnosis and surgical management. J Oral Implantol. 2014;40(2):189-95. https://doi.org/10.1563/AAID-JOI-D-12-00011. | 3 |
| **51.** | Mermer RW, Rider CA, Cleveland DB. Nasopalatine canal cyst: a rare sequelae of surgical rapid palatal expansion. Oral Surg Oral Med Oral Pathol Oral Radiol Endod. 1995;80(6):620. https://doi.org/10.1016/s1079-2104(05)80238-0. | 2 |
| **52.** | Minami K, Sakamoto S, Nishimura K, Ichimaru K, Sato S, Haji T. A Case of a Nasopalatine Duct Cyst Surgically Opened to the Bilateral Nasal Cavity. Prac Oto-Rhino-Laryngol. 2013;106(3):207-12. | 1 |
| **53.** | Nagaraj T, Gogula S, Sumana CK, Nigam H. Incisive canal cyst-A case report. Int J Med Dent Case Rep. 2017;4:1-2. | 1 |
| **54.** | Naini FB, Aminishakib P, Ghorbanpour M, Vakili MM, Kharazifard MJ. Demographic Profile of Non-Odontogenic Jaw Lesions in an Iranian Population: A 30-Year Archive Review. J Dent (Tehran). 2017;14(3):132-137. | 3 |
| **55.** | Nelson BL, Linfesty RL. Nasopalatine duct cyst. Head Neck Pathol. 2010;4(2):121-2. https://doi:org/10.1007/s12105-010-0169-3. | 3 |
| **56.** | Nortjé CJ, Farman AG. Nasopalatine duct cyst. An aggressive condition in adolescent Negroes from South Africa? Int J Oral Surg. 1978;7(2):65-72. https://doi.org/10.1016/s0300-9785(78)80049-0. | 3 |
| **57.** | Nortjé CJ, Wood RE. The radiologic features of the nasopalatine duct cyst. An analysis of 46 cases. Dentomaxillofac Radiol. 1988;17(2):129-32. https://doi.org/10.1259/dmfr.1988.0017. | 3 |
| **58.** | Oda T, Sue M, Sasaki Y, Ogura I. Diffusion-weighted magnetic resonance imaging in oral and maxillofacial lesions: preliminary study on diagnostic ability of apparent diffusion coefficient maps. Oral Radiol. 2018;34(3):224-8. https://doi.org/10.1007/s11282-017-0303-y. | 2 |
| **59.** | Ogura I, Nakahara K, Sasaki Y, Sue M, Oda T. Diffusion-weighted Magnetic Resonance Imaging in Odontogenic Keratocysts: Preliminary Study on Usefulness of Apparent Diffusion Coefficient Maps for Characterization of Normal Structures and Lesions. Chin J Dent Res. 2019;22(1):51-56. https://doi.org/10.3290/j.cjdr.a41775. | 1 |
| **60.** | Ojl C. Statistical observations on jaw cysts in Enugu, Nigeria, 1987-1996. Trop Dent J. 1999;33-6. | 2 |
| **61.** | Orosz M, Ferenczi I, Szende B. Nagyméretu unilaterális canalis incisivus cysta ritka esete [Rare case of the large-sized, unilateral incisive canal cyst]. Fogorv Sz. 2004;97(5):195-7. | 1 |
| **62.** | Panjwani S, Arora S, Rai S, Malik R. Role of cone beam computed tomography in the prompt diagnosis of a nasopalatine duct cyst. J Indian Acad Oral Med Radiol. 2014;26:338-41. https://doi.org/10.4103/0972-1363.145023. | 3 |
| **63.** | Parwani R, Parwani S, Wanjari S. Diagnosis and management of bilateral nasolabial cysts. J Oral Maxillofac Pathol. 2013 Sep;17(3):443-6. https://doi.org/10.4103/0973-029X.125217. | 1 |
| **64.** | Perez A, Lenoir V, Lombardi T. Aggressive Nasopalatine Cyst with Nasal Involvement in an Edentulous  Patient. Appl. Sci. 2022; 12(11002):1-6. https://doi.org/10.3390/app122111002. | 3 |
| **65.** | Perumal JC. An Unusually Large Destructive Nasopalatine Duct Cyst: A Case Report. J. Maxillofac. Oral Surg. 2013;12(1):100–4. https://doi.org/10.1007/s12663-011-0201-5. | 3 |
| **66.** | Pevsner PH, Bast WG, Lumerman H, Pivawer G. CT analysis of a complicated nasopalatine duct cyst. N Y State Dent J. 2000;66(6):18. | 3 |
| **67.** | Póvoa RCS, Braga CLS, Telles L, Raia ELRM, Braune AS, Homsi N, Cardoso ES, Cardoso AS. Oralnasal fistula repair after enucleation of nasalpalatine duct cyst. Int J Oral Maxillofac Surg. 2019;48:66-7. | 2 |
| **68.** | Popli H, Singh H, Gupta A, Kamboj M. Peripheral Ossifying Fibroma Veiling a Nasopalatine Duct Cyst:  An Unusual Concurrence. Indian J Otolaryngol Head Neck Surg. 2022;74(Suppl2):S1459–61. https://doi.org/10.1007/s12070-021-02601-0. | 3 |
| **69.** | Rapidis AD, Langdon JD. Median cysts of the jaws--not a true clinical entity. Int J Oral Surg. 1982;11(6):360-3. https://doi.org/10.1016/s0300-9785(82)80059-8. | 2 |
| **70.** | Redman RS. Nasopalatine duct cyst with pigmented lining suggestive of olfactory epithelium. Oral Surg Oral Med Oral Pathol. 1974;37(3):421-8. https://doi.org/10.1016/0030-4220(74)90115-7. | 3 |
| **71.** | Ricucci D, Amantea M, Girone C, Feldman C, Rôças IN, Siqueira Jr JF. An unusual case of a large periapical cyst mimicking a nasopalatine duct cyst. J Endod. 2020;46(8):1155-62. | 1 |
| **72.** | Robertson H, Palacios E. Nasopalatine duct cyst. Ear Nose Throat J. 2004;83(5):313. | 3 |
| **73.** | Rodrigues ML, Maia RS, Neves Neto BF, Cruz VM, Horiuchi ZHFN, Horiuchi NCF. Surgical removal of odontoma and nasopalatine duct cyst in the maxilla region: case report. RSBO. 2023;20(2):494-500. | 1 |
| **74.** | Sankar D, Muthusubramanian V, Nathan JA, Nutalapati RS, Jose YM, Kumar YN. Aggressive nasopalatine duct cyst with complete destruction of palatine bone. J Pharm Bioallied Sci. 2016;8(Suppl 1):S185-8. https://doi.org/10.4103/0975-7406.191956. | 3 |
| **75.** | Sarangi S, Ray D, Bhattacharjee T, Ray JG. A brief insight regarding Nasopalatine duct cyst- Report of two cases with a review of literature. J Oral Maxillofac Pathol. 2024;28(3):483-487. https://doi.org/10.4103/jomfp.jomfp_3_24. | 3 |
| **76.** | Saunders LA, Wisniewski H, Soumerai S. Extensive incisive canal cyst. Report of a case. Oral Surg Oral Med Oral Pathol. 1968;26(3):284-90. https://doi.org/10.1016/0030-4220(68)90396-4. | 3 |
| **77.** | Scolozzi P, Martinez A, Richter M, Lombardi T. A nasopalatine duct cyst in a 7-year-old child. Pediatr Dent. 2008;30(6):530-4. | 3 |
| **78.** | Sebastian S, Pandiar D, Krishnan RP. Nasopalatine Duct Cyst: A Clinicopathological Analysis of Ten New Cases from a Tertiary Oral Health Care Centre. J Maxillofac Oral Surg. 2025;24:1827-36. https://doi.org/10.1007/s12663-025-02726-7 | 3 |
| **79.** | Shakib K, McCarthy E, Walker DM, Newman L. Post operative maxillary cyst: report of an unusual presentation. Br J Oral Maxillofac Surg. 2009;47(5):419-21. https://doi.org/10.1016/j.bjoms.2008.09.016. | 2 |
| **80.** | Shirabe K. Incisive canal cyst. Especially its operative method. Otologia Fukuoka. 1973;19(5):633-7. | 2 |
| **81.** | Shylaja S, Balaji K, Krishna A. Nasopalatine Duct Cyst: Report of a Case with Review of Literature. Indian J Otolaryngol Head Neck Surg. 2013;65(4):385-8. https://doi.org/10.1007/s12070-011-0242-6. | 3 |
| **82.** | Srikanth G, Krishnan R, Malladi UK, Shetty N, Carnelio S, Shukla AD. Nasopalatine Duct Cyst Associated with Non-Vital Teeth: Report of a Rare Case. Indian J Otolaryngol Head Neck Surg. 2024;76(3):2761-4. https://doi.org/10.1007/s12070-024-04513-1. | 3 |
| **83.** | Srivastava S, Misra N, Agarwal R, Pandey P. Nasopalatine canal cyst: often missed. BMJ Case Rep. 2013. https://doi.org/10.1136/ bcr-2012-007548. | 3 |
| **84.** | Stam FC, van der Waal I, van der Kwast WAM. Pigment in the lining of nasopalatine duct cysts: Report of two cases. J Oral Pathol Med. 1979;8(3):170-175. https://doi.org/10.1111/j.1600-0714.1979.tb01823.x | 2 |
| **85.** | Strickland M, Singer SR, Rinaggio J, Kim IH, Mupparapu M. Large, expansile odontogenic cyst with bilateral maxillary sinus involvement. N Y State Dent J. 2013 Mar;79(2):38-40. | 1 |
| **86.** | Suter VG, Sendi P, Reichart PA, Bornstein MM. The nasopalatine duct cyst: an analysis of the relation between clinical symptoms, cyst dimensions, and involvement of neighboring anatomical structures using cone beam computed tomography. J Oral Maxillofac Surg. 2011;69(10):2595-603. https://doi.org/10.1016/j.joms.2010.11.032. | 3 |
| **87.** | Suter VG, Warnakulasuriya S, Reichart PA, Bornstein MM. Radiographic volume analysis as a novel tool to determine nasopalatine duct cyst dimensions and its association with presenting symptoms and postoperative complications. Clin Oral Investig. 2015;19(7):1611-8. https://doi.org/10.1007/s00784-014-1391-2. | 3 |
| **88.** | Suter VG, Jacobs R, Brücker MR, Furher A, Frank J, von Arx T, Bornstein MM. Evaluation of a possible association between a history of dentoalveolar injury and the shape and size of the nasopalatine canal. Clin Oral Investig. 2016;20(3):553-61. https://doi.org/10.1007/s00784-015-1548-7. | 1 |
| **89.** | Swanson KS, Kaugars GE, Gunsolley JC. Nasopalatine duct cyst: an analysis of 334 cases. J Oral Maxillofac Surg. 1991;49(3):268-71. https://doi.org/10.1016/0278-2391(91)90217-a. | 3 |
| **90.** | Takagi R, Ohashi Y, Suzuki M. Squamous cell carcinoma in the maxilla probably originating from a nasopalatine duct cyst: report of case. J Oral Maxillofac Surg. 1996;54(1):112-5. https://doi.org/10.1016/s0278-2391(96)90318-3. | 1 |
| **91.** | Takeshita K, Funaki K, Jimbo R, Takahashi T. Nasopalatine duct cyst developed in association with dental implant treatment: A case report and histopathological observation. J Oral Maxillofac Pathol. 2013;17(2):319. https://doi.org/10.4103/0973-029X.119761. | 3 |
| **92.** | Tanaka S, Iida S, Murakami S, Kishino M, Yamada C, Okura M. Extensive nasopalatine duct cyst causing nasolabial protrusion. Oral Surg Oral Med Oral Pathol Oral Radiol Endod. 2008;106(4):e46-50. https://doi.org/10.1016/j.tripleo.2008.05.046. | 3 |
| **93.** | Terry BR, Bolanos OR. A diagnostic case involving an incisive canal cyst. J Endod. 1989;15(11):559-62. https://doi.org/10.1016/S0099-2399(89)80202-X. | 3 |
| **94** | Thoma KH. Case No. 48. Incisive canal cyst. Am J Orthod Oral Surg. 1941;27(4):A226-8. | 2 |
| **95.** | Tsuneki M, Maruyama S, Yamazaki M, Abé T, Adeola HA, Cheng J, Nishiyama H, Hayashi T, Kobayashi T, Takagi R, Funayama A, Saito C, Saku T. Inflammatory histopathogenesis of nasopalatine duct cyst: a clinicopathological study of 41 cases. Oral Dis. 2013;19(4):415-24. https://doi.org/10.1111/odi.12022. | 3 |
| **96.** | Vasconcelos R, de Aguiar MF, Castro W, de Araújo VC, Mesquita R. Retrospective analysis of 31 cases of nasopalatine duct cyst. Oral Dis. 1999;5(4):325-8. https://doi.org/10.1111/j.1601-0825.1999.tb00098.x. | 3 |
| **97.** | Wu YH, Wang YP, Kok SH, Chang JY. Unilateral nasopalatine duct cyst. J Formos Med Assoc. 2015;114(11):1142-4. https://doi.org/10.1016/j.jfma.2015.08.005. | 2 |
| **98.** | Yeom HG, Kang JH, Yun SU, Yoon JH. Nasopalatine duct cyst with sebaceous differentiation: a rare case report with literature review. BMC Oral Health. 2021;21(1):419. https://doi.org/10.1186/s12903-021-01772-0. | 3 |
| **99.** | Yüksel H, Büyük B, Evlice B. -Diagnosis of Nasopalatine Duct and Nasopalatine Duct Cyst in CBCT Images: A Radiomics-Based Machine Learning Approach. Dentomaxillofac Radiol. 2025:twaf076. https://doi.org/10.1093/dmfr/twaf076. | 2 |
| **100.** | Yusa K, Kasuya S, Sasahara N, Hemmi T, Ishikawa S. A Rare Case of Nasopalatine Duct Cyst Diagnosed 7 Years After Secondary Bone Grafting in a Patient With Cleft Lip and Palate. Case Rep Dent. 2025;2025:5520791. https://doi.org/10.1155/crid/5520791. | 3 |

Reasons for exclusion: 1- Reported pathology is not NDC; 2- Study design (Literature reviews, experimental studies, short communications, letters to the editor, in vitro and ex vivo studies); 3- Duplicate from main databases.
